# The role of Th-17 cells and IL-17 in the metastatic spread of breast cancer: As a means of prognosis and therapeutic target

**DOI:** 10.3389/fimmu.2023.1094823

**Published:** 2023-03-13

**Authors:** Tewodros Shibabaw, Banchamlak Teferi, Birhanu Ayelign

**Affiliations:** ^1^ Department of Biochemistry, School of Medicine, College of Medicine and Health Sciences, University of Gondar, Gondar, Ethiopia; ^2^ Department of Clinical Pharmacy, School of Pharmacy, College of Medicine and Health Sciences, University of Gondar, Gondar, Ethiopia; ^3^ Department of Immunology and Molecular Biology, School of Biomedical and Laboratory Science, College of Medicine and Health Science, University of Gondar, Gondar, Ethiopia; ^4^ Research School of Biology, College of Science, Australian National University, Canberra, ACT, Australia

**Keywords:** bone metastasis, breast cancer, inflammation, antitumor effects, MAPK, NF-kB, MMPs, interleukin-17A

## Abstract

Metastatic breast cancer is one of the most common and well-known causes of death for women worldwide. The inflammatory tumor cell and other cancer hallmarks dictate the metastatic form and dissemination of breast cancer. Taking these into account, from various components of the tumor microenvironment, a pro-inflammatory infiltrative cell known as Th-17 plays an immense role in breast cancer proliferation, invasiveness, and metastasis. It has been demonstrated that IL-17, a pleiotropic pro-inflammatory cytokine generated by Th-17, is upregulated in a metastatic form of breast cancer. Recent research updates stated that chronic inflammation and mediators like cytokines and chemokines are causative hallmarks in many human cancers, including breast cancer. Therefore, IL-17 and its multiple downward signaling molecules are the centers of research attention to develop potent treatment options for cancer. They provide information on the role of IL-17-activated MAPK, which results in tumor cell proliferation and metastasis *via* NF-kB-mediated expression of MMP signaling. Overall, this review article emphasizes IL-17A and its intermediate signaling molecules, such as ERK1/2, NF-kB, MMPs, and VEGF, as potential molecular targets for the prevention and treatment of breast cancer.

## Introduction

Breast cancer (BC) is the most frequently diagnosed metastatic cancer among women (1). It is a molecularly diverse disease that involves complex processes that result in initiation, progression, and metastasis (2). The tumors of breast origin can be classified either by the gene expression pattern of three receptors, such as estrogen receptor (ER), progesterone receptor (PR), and human epidermal growth factor receptor 2 (HER2), or through its nodal metastasis (3). Like other solid tumors, breast cancer starts locally and spreads into distant organs—metastatic breast cancer (2). Breast cancer metastasis is a usual hallmark of cancer and leads to treatment failure, leading to the death of many patients. Around 10%–15% of breast cancer patients experience metastasis, leading to death (2). The bone is the most common site of breast cancer metastasis for about 75% of patients with late-stage BC (4). Usually, metastatic breast cancer has a poor prognosis, with 73% of the patients having less-than-5-year survival (5, 6). According to studies, several factors may affect the pathogenesis and prognosis of breast cancer (6). The genetic mutation of tumor cells is responsible for the proliferation, uncontrolled growth, and spreading ability of the primary tumor cell malignancy invasiveness and distant migration (7–9). Inflammatory condition is also a prognostic factor in metastatic breast cancer and contributes to cancer development and progression. Particularly, immune cells—including Th-17, tumor-associated macrophage, neutrophils, natural killer (NK) cells, and γδT cells and mediators in the microenvironment—facilitate angiogenesis and proliferation (10–12). Th-17 is one of the inflammatory CD4+ cells that play an essential role in cancer pathogenesis and anti-tumor immune response (13). Notably, in breast cancer, Th-17 cells are positively related to IL-6, IL-1β, and IL-17 expression and negatively correlated with increased metastatic lymph nodes and tumor cell angiogenesis. IL-17-induced inflammatory mediators such as G-CSF, IL-6, and CXCL1 stimulate the expansion and recruitment of dysfunctional myeloid cells to establish a proangiogenic and immune-suppressive tumor environment that enhances tumor growth and metastasis (14). This results in the formation of a metastatic secondary tumor. However, how the cytokine of the microenvironment promotes tumor metastasis remains a research question. For the effective migration and metastasis of breast cancer cells in the vascular or lymphatic drainage system, chemical mediators such as calcium-dependent zinc-containing endopeptidases like MMPs must be required for the degradation of the ECM as well as VEGF and IL-8 for vascularization during intravasation and extravasation processes, respectively, and reach to the bone (15). A study showed that the expression and activation of MMPs are mediated through TNF-α and IL-1 secreted by tumor cells, and IL-17A secreted from the microenvironment plays a role on the regulation of different MMPs (16, 17). There are five major classes of MMPs depending on their function and the substrates that they digest, including matrilysins (MMP-7 and MMP-26), collagenases (MMP-1, MMP-8, and MMP-13), stromelysins (MMP-3, MMP-10, and MMP-11), gelatinase (MMP-2 and MMP-9), and membrane-associated metalloproteinases (MMP-14, MMP-15, MMP-16, MMP-17, MMP-23A/B, MMP-24, and MMP-25) (18, 19). A retrospective SEER study on 25,323 women presenting with stage IV BC explored that 26.8% and 12.8% had overall survival of 5 and 10 years, respectively (20, 21). It explained that there is a strong association between elevated rates of IL-17 and Th-17 cell infiltration and estrogen receptor (ER)-negative and triple-negative molecular subtypes of BC (22).

In the mammary gland tumor microenvironment, excessive infiltration of Th-17 cells, NK, and γδT cells are associated with poor prognostic factors for staging, overall, and disease-free survival (23, 24). Furthermore, the functional contribution of human Th-17 cells to tumor immunity remains unclear since both pro- and anti-tumor effects have been observed. According to the data, in both the 4T1 and E0771 tumor models, increased Th-17 was seen at the early stage of tumor progression, peaked at the middle cancer stage, and then markedly declined at the late stage (25). This review further elaborates on the interaction of IL-17A with its heterodimer single-pass transmembrane receptor (IL17RA/IL17RC). In turn, TRAF-6/TAK-1 joins the MAPK pathway and upregulates the subsequent phosphorylation of extracellular signal-regulated kinases (ERK1/2) in all human breast cancer, thus leading to uncontrolled growth, proliferation, and resistance to traditional chemotherapeutic agents such as docetaxel (26). In addition, these reviews will discuss the IL-17/NF-kB-associated incidence of bone metastatic breast cancer. The progression and metastasis of BC thought to be controlled through locally infiltrated Th-17 cells produce inflammatory cytokines (IL-17A). This results from activating the IL-17A-IL-6-STAT-3 pathways, NF-kB-mediated production of MMPs, and vascular endothelial cell growth factor (VEGF) (27). In supporting the abovementioned scenario0, IL-17A also triggers the growth and proliferation of tumor cells through the IL-17A/MAPK pathways (14, 28). Overall, following metastasized breast cancer diagnosis, biological signaling pathways are the foundation of current anti-cancer therapies. Therefore, it is crucial to thoroughly understand the molecular and immunological mechanisms to classify and design appropriate treatment for breast cancer (29). This review article aimed to illustrate the dysregulated MAPK and NF-kB pathways in response to IL-17A/IL-17AR/CR interaction in bone metastatic breast cancer and its therapeutic options.

## Mechanism of Th-17 cell polarization and the diversity of IL-17 and IL-17R

**Figure 1:**
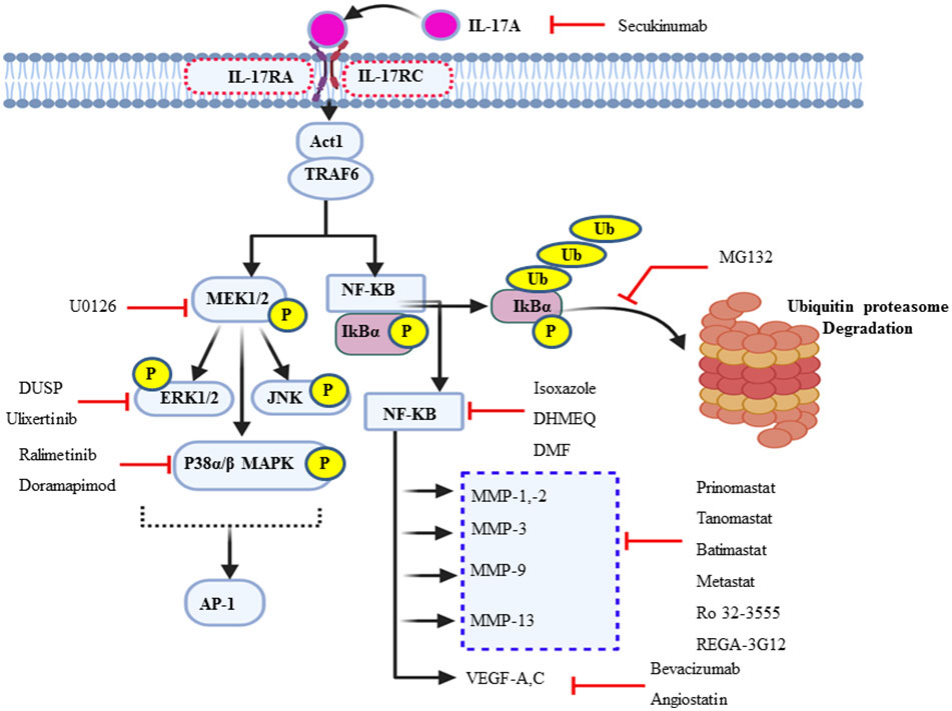
Schematic illustration of Th-17 polarization and its cytokine signature. Chemokines, including CCL2 and MCP-1, are secreted by cancer-associated fibroblasts (CAFs), tumor-associated macrophages (TAM), and antigen-presenting cells (APC) that promote CD4+ cell recruitment. Breast cancer cells, CAFs, TAMs, and dendritic cells are part of the stem cell and its microenvironment that produces different polarizing cytokines, such as IL-6, IL-23, and TGF-β. In turn, such polarizing cytokines activate distinct transcription factor cascades within naïve CD4+ T cells and influence T cell differentiation into distinct effector T cell subtypes, mainly Th-17 effector cell subset that produces all IL-17 signature cytokines (IL-17A, IL-17B, IL-17C, IL-17D, IL-17E, and IL-17F) and IL-21 and IL-22.

The third independent lineage of the CD4^+^ T cell subset, designated as “Th-17 cell”, produces IL-17A, and a related family of IL-17 cytokines was discovered in 2005 (30–33). Currently, there are six related IL-17 family members such as IL-17A, IL-17B, IL-17C, IL-17D, IL-17E, and IL-17F (30, 34, 35). Both IL-17F and IL-17A share the same structural similarities and are secreted in homodimeric (two IL-17A or two IL-17F) or heterodimeric (IL-17A/IL-17F) forms. These forms are biologically active and connected by disulfide bonds formed by its cysteine residues (36). Further studies have shown that “Th-17” cells are also capable of secreting IL-21, IL-22, and GM-CSF (37). IL-21 creates an amplification loop for the further generation of Th-17 cells (38), whereas IL-17A is mostly secreted as a heterodimeric form with IL-17F, suggesting that the activity of IL-17A is partially attributed to the most potent form of IL-17A/IL-17F heterodimeric cytokine (36, 39)., Apart from Th-17, IL-17 can also be secreted by γδT cells, natural killer (NK) cells, NK T cells, mast cells, granulocytes, a subset of CD8+ T cells, known as Tc17 cells, and “innate lymphoid cells” (35, 40). The tumor cells, breast cancer-associated macrophage (41), and cancer-associated fibroblasts (CAF) secrete chemokines such as MCP-1 or CCL2, CCL20 (MIP-3A) (42, 43), and CXCL12 (SDF-1) as chemo-attraction of CD4+ T cell during differentiation of Th-17 cell and even for the selective attraction of Th-17 cell infiltration and/or its migration into the site of the tumor microenvironment (28). The ability of naive CD4+ T cells to undergo lineage polarization into distinct effector subsets is mediated by master transcription factors (44). These master transcription factors play opposing roles in Th-1/Th-2/Th-17/Treg cell fate decisions; retinoic acid receptor-related orphan receptor-γt (ROR-γt) is induced during Th-17 cell differentiation and strongly suppresses other Th cell polarization of the gene expression (45). Downstream of STAT3 signaling is the Th-17 master regulator ROR-γt. This transcription factor directly regulates the expression of IL-17A and IL-17F, along with other Th-17-specific genes (44, 46). Therefore, the polarization of naïve CD4+ T cell to Th-17 cells takes two significant steps in the reaction process. Activation of naïve CD4+ T cell is the first step of the reaction, mediated by cytokines (IL-6, IL-23, and TGF-β) secreted by a professional APC such as dendritic cells and macrophages (**Figure 1**) (30, 47). Taken together, polarizing cytokines produced by APC of the tumor microenvironment and cancer stem cells are the driving force for differentiation, maturation, and survival (IL-23 mediated) of the Th-17 phenotype (48, 49). In the context of inflammation driven by cytokines such as tumor necrosis factor (TNF), there is a clear synergy with IL-17F, reaching a pro-inflammatory gene signature very far from that induced by the combination of TNF-α and IL-17A (50). Depending on amino acid homology with IL-17A, the remaining related families, such as IL-17C, IL-17D, and IL-17E, have been identified and are significantly divergent from IL-17A (51). Research scholars stated that the inflammatory potency of IL-17F becomes more amplified when expressed and works together with tumor necrosis factor (TNF-α) (52). The signal transduction of each member of the IL-17 family is through its binding to specific interleukin-17 receptors (IL-17R) (34). The tissue distribution of IL-17RAs is almost in every cell type, whereas IL-17RC is predominantly expressed in epithelial cells, endothelial cells, fibroblasts, osteoblasts, and limited expression in myeloid cells (53–55). Based on sequence homology to IL−17RA, additional receptors have been identified in the IL−17R family such as IL−17RB, IL−17RC, IL−17RD, and IL−17RE. Indeed not only IL−17RA but also IL−17RC is required for the action of both IL−17A and IL−17F. Therefore, it showed that IL-17A or IL-17F binds the receptor complex called IL-17RA–IL-17RC to drive the expression of a gene involved in the inflammation, proliferation, angiogenesis, and metastasis of primary tumor cells through NF-kB and MAPK activation (56–58).

## IL-17A/MAPK signaling promotes the proliferation of breast cancer cell

**Figure 2:**
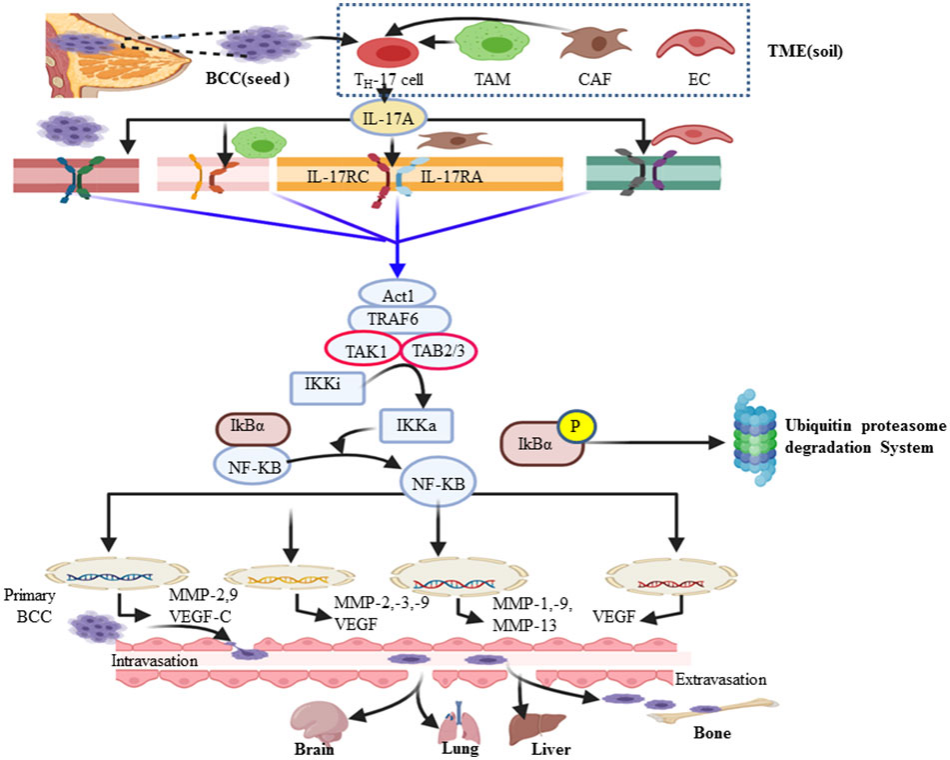
An overview of the IL-17A/MAPK signaling pathway in the proliferation of breast cancer. ERK1/2, JNK, and p38 MAPK are the major effector molecules of this signal cascade and lead to phosphorylate multiple transcription factors, particularly activator protein one (AP-1), which is a hetero-dimeric composition of *c-Jun* and *c-Fos* proteins. Once active, it translocates to the nucleus and orchestrates the expression and function of many proliferative genes or cell cycle regulators such as cyclin D1 (G1-to-S phase) and cyclin A/E (S-to-G2 phase). In turn, it increases cell growth or proliferation and survival.

IL-17 plays a pivotal role in the tumor microenvironment, from the initial stages of tumorigenesis to its invasiveness, proliferation, and distant migration (14). Therefore, after the dysregulated interaction of IL-17 with its receptor, there will be a feed-forward expression of other inflammatory molecules, such as IL-6 through NF-kB. In turn, IL-6 joins the vicious cycle or loop *via* the additional activation of NF-kB through the IL-6/STAT-3/NF-kB axis (14). A study on a variety of BC cell line in a mouse model explored that IL-17 has a pro-tumoral effect and contributes to chemotherapeutic resistance (for example, paclitaxel). IL-17A/E induces c-RAF and ERK1/2 phosphorylation by p70S6 kinase; in turn, it activates the MAPK signaling pathway and contributes to BC taxane resistance. All IL-17R receptors contain an extracellular domain (ligand binding site), a transmembrane domain, and a cytoplasmic domain “SEFIR” (56). The SEFIR domain of IL-17RA is a conserved cytoplasmic motif that depends on an adapter molecule ACT1 (also known as TRAF3IP2) and TNF receptor−associated factor 6 (TRAF6) to recruit the rest of its downstream signalings such as MAPK (p38, JNK, and ERK1/2) (59, 60) and the NF-kB pathway (**Figure 2**) (34) (61–63). Taking this step further, ACT1 recruitment relays on the SEFIR domain, then TARF-6 binds to ACT-1 and form the IL-17R complex (64). In addition, ACT-1 also plays a non-degradative ubiquitination of TRAF-6 through its U-box, like lysine-63 (K63) E3 ligase domain (32). In turn, the ubiquitination of TRAF6 provides a scaffold for the recruitment and activation of TAK-1 (MAPKKK)-mediated activation of MEK-1/2 (65). Elevated IL-17A or IL-17B expression is strongly associated with poor prognosis outcomes for patients who present with BC (22, 66). To stimulate cells to proliferate or differentiate, these short-lived signaling events need to be converted into longer-lasting ones that can sustain the signal and relay it downstream to the nucleus (67). ERK (ERK1 and ERK2) is activated upon phosphorylation by MEK (MEK1 and MEK2), which is itself activated when phosphorylated by TAK-1 (**Figure 2**). In support of this, studies showed that the type of IL-17 response is context dependent, *i*.*e*., different Il-17 families affect the cell with different effector molecules differently concerning cell lines like MCF7, T47D, BT20, MDA-MB468, MD-MB157, and MDA-MB231 (**Table 1**) (68, 69). Few preclinical studies support the antitumorigenic properties, particularly of IL-17-E. In contrast, many more clinical as well as preclinical studies explained pro-tumorigenesis with the exposures of IL-17A, IL-17F, and IL-17B (75). Moreover, similar to that of the overexpression or mutation of receptor tyrosine kinase, most cancers, including breast cancer-associated lesions, that lead to constitutive or uncontrolled activation of ERK signaling (55) were due to either the overexpression of IL-17A/IL-17RA and ACT1-TRAF-6 or the activating mutation of TAK-1 molecule (63, 76, 77). However, there is also amplification or deregulation of its nuclear transcription factor targets, such as AP-1 (dimeric transcription factor of c-Fos and c-Jun) (78). AP-1 does not always promote cell proliferation but has anti-proliferative activities (79). In turn, activated c-Jun-containing AP-1 allows positive G_1_-to-S-phase progression, proliferation, and differentiation regulators such as cyclin D1 to be turned on and transcribed (80) or represses or turns off tumor suppressor p53 (blocker of CD1 and cyclin A/E *via* P21) and p16 expression (81–83). Additionally, the study showed that IL-17A is produced by BC TILs and responsible for docetaxel chemoresistance, angiogenesis, and its proliferation potential through the ERK1/2 pathway and induction of phosphorylation of EGFR in collaboration with IL-17ER (69). It proposed that multi-target inhibition, *i*.*e*., not only targeting IL-17A/IL-17AR/CR but also inhibiting its co-worker IL17E/IL-17ER, maximizes the clinical efficiency or potency of anti-EGFR such as panitumumab or rrastuzumab for treatments of BC (84, 85). Among MAPK signaling, p38 and ERK1/2 are the most commonly activated in tumorigenesis and migration of BC (86–88). During BC cell proliferation and invasion, IL-17 is suggested to be critical for p38 MAPK activation. The activated p38 MAPK, in turn, promotes the production of cytokines (TGFβ and TNFα) and interleukins (IL-6, IL-8, and IL-1β) within the tumor microenvironment, all of which are known to play a role in promoting tumor growth, angiogenesis, invasion, and metastasis (89).

**Table 1:** *In vivo* and *in vitro* roles of IL-17 cytokines in breast cancer.

Types of cytokines	Cellular source	Types of study	Cell lines	Response on IL-17 exposure	Cellular mechanisms	References
IL-17A	Th-17, TAM, CAF	Preclinical and clinical	MCF7, T47D, BT20, MDA-MB468, MD-MB157, MDA-MB231	Pro- tumorigenesis	Activation or ERK1/2 pathway induces proliferation, migration, invasion, and chemoresistance Recruitment of macrophages, activation of MMP	(68, 69)
IL-17A	Th-17, TAM, CAF	Clinical	None	Pro- tumorigenesis	IL-17A associated to MMP-1, 2, 3, 9, and 11 mononuclear infiltrating cells which are correlated to metastasis	(70)
IL-17A	Th-17, TAM, CAF	Preclinical	MCF7	Pro- tumorigenesis	Activation of MAPK: MEKK, ERK, JNK, cJun, STAT3 Cell proliferation	(71)
IL-17E	Th-17	Preclinical	MCF7, MDA-MB468, MDA-MB 435-S, MDA-MB231, SKBR3, T47D, ZR75, Hs578t, HCC1937, MDA-MB175-7	Anti- tumorigenesis	Induction of apoptosis, decrease in colony formation and tumor growth	(72, 73)
IL-17A and IL-17E	Th-17 cell,	Preclinical and clinical	47D, MCF7, BT20, IJG-1731	Pro- tumorigenesis	Activation of cRAF and S6 kinases and *via* chemoresistance	(73)
IL-17B	Th-17 cell	Preclinical and clinical	MCF7, MDA-MB-157, MDA-MB-231, MDA-MB-361, BT20	Pro- tumorigenesis	Resistance to paclitaxel in cell lines *via* ERK pathway Upregulation of BCL2 promotion of proliferation and tumor growth through IL-17RB *via* NF-kB and TRAF6	(74)
IL-17E	Th-17 cell	Preclinical study	MCF7, MDA-MB468, MDA-MB 435-S, MDA-MB231, SKBR3, T47D, ZR75, Hs578t, HCC1937, MDA-MB175-7	Anti- tumorigenesis	Induction of apoptosis, decrease in colony formation and tumor growth	(72, 75)

## IL-17A//NF-kB/MMPs axis promotes bone metastatic breast cancer

**Figure 3:**
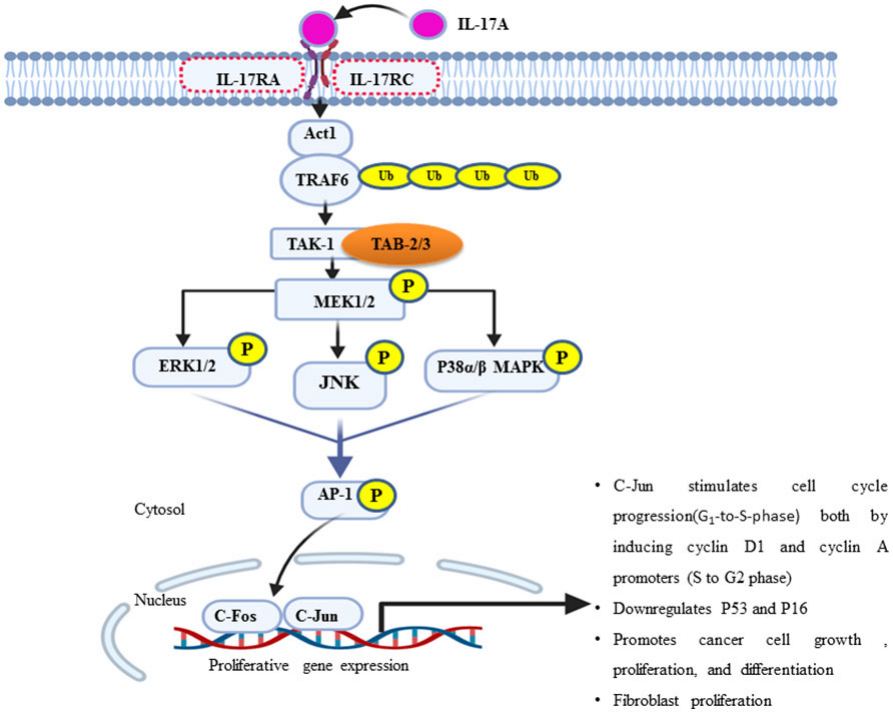
Diagram summarizing the mechanism of the IL-17A/NF-kB pathway in breast cancer metastasis and other organs like the brain. IL-17A can be secreted by breast cancer (BC) cells and many cells in the breast cancer microenvironment such as tumor-associated macrophages, cancer-associated fibroblasts, Th-17 cell, γδT cells, and endothelial cells. In turn, IL-17 and other Th-17 derived cytokines influence the tumor microenvironment by directly promoting transformed cell properties and the nearby stromal cell activity. IL-17A binds with IL-17RA–IL-17RC receptor and transduces signaling *via* the adaptor protein nuclear factor (NF)-k activator (Act1). Many IL-17 target genes contain the promoter’s region that binds with NF-kB. In turn, the NF-kB signal pathway tend to be activated and promote the expression of genes encoded for angiogenesis and metastasis. The major steps of bone metastatic BC include extravasation, circulatory journey, extravasation in distant sites, and ultimately metastatic colonization of bone or other target organs (brain, lung, and liver).

Upon the interaction of IL-17A with its corresponding receptor, the u-box domain of Act1 is essential for IL-17-induced NF-kB activation (90). ACT-1-mediated ubiquitination of TRAF-6 acts as a scaffolding intermediate of the IL-17A signaling pathway (40). TRAF6 is also a signaling adaptor molecule that plays a key role as an E3 ubiquitin ligase and ubiquitin-conjugating enzyme (E2) complex composed of Ubc13 and Uev1A (91). Subsequently ubiquitinated TRAF-6 recruits a protein kinase complex involving TGF-β-activated kinase 1 (TAK1) and TAK1-binding proteins (TAB2–TAB3) (60). TAK1, a member of the MAP kinase kinase (MAP3K) family, then activates the inactive IkB kinase (IKKi) complex (IKKα/β/γ) into its activated form (IKKa) *via* phosphorylation (92). In turn, IKKa then phosphorylates the IκB subunit of the NF-κB/IκB complex, marking IκB for E3 ubiquitin ligase–proteasomal proteolysis (42, 43). Ubiquitin (Ub) itself can be further ubiquitinated and form a polyubiquitin (poly-Ub) chain on IκB. Then, IκB becomes recognizable by the proteasome. Ub–proteasome-based degradation of IκB makes NF-kB free of it, translocates to the nucleus, and acts on a wide spectrum of the NF-kB gene response element involved in the inflammation and metastasis of cancer (**Figure 2**) (93). The major cause of cancer-associated morbidity and mortality is its metastasis and colonization of other organs like bone, lung, liver, and brain in the case of BC (94, 95). Thus, cancer develops after migration to other anatomic sites, which are called secondary tumors (96). NF-kB key transcription factor plays a role in the expression and activity of MMPs (16, 17, 97). This, in turn, defines as many of the effects of IL-17A that are correlated with the TRAF-6-mediated activation of NF-kB. Therefore, the NF-kB-mediated expression of MMP-2 and -9, respectively, are the most important driving force in the invasiveness and metastasis of various human cancers such as colorectal cancer (98), hepatocellular cancer (99), nasopharyngeal carcinoma (100), and non-small cell lung cancer (101). Similarly, the researchers explored that the NF-kB-mediated invasiveness, migration, and metastasis of BC also rely on the increased expression of MMP-2, MMP-13, MMP-9, and MMP-1 (**Figure 3**) (102–104). MMP-13, known as collagenase-3, plays in ECM physical barrier degradation and increases the invasive capacities of the malignant cells (70). In support of this, MMP-13 mRNA and its protein expression in BC serve as independent biomarkers of poor prognosis or shorter overall survival (95,) (105). In addition, tumor-associated macrophage (M2Ф) secretes MMP-13 and MMP-3 which are involved in the promotion of metastasis *via* the IL-17/IL-8 axis (105–107). Similar to M2Ф, the CAF cells of the microenvironment also secretes MMP-1, MMP-11, MMP-9, and MMP-13 (**Figure 3**). In the clinical diagnosis of high-grade (grade-3) breast cancer, the study showed exuberantly increases MMP-2 and MMP-9 mRNA and protein expression (19, 108). Furthermore, several other studies support a pro-tumorigenesis role of IL-17 in BC, and the details are presented in **Table 1**. Those studies also elaborate that the level of IL-17 was increased and correlated with the expansion of the disease. Moreover, p38/NF-kB-mediated transcription products such as TNF-α, MMPs (MMP-2 and MMP-9), VEGF (also called VEGF-A and located at chromosome 6p12), VEGF-C, and PGE1/2 facilitate the invasion and metastasis of cancer (34, 41,) (109). Judah Folkman (father of angiogenesis) stated in 1974 that no tumor could grow beyond 2 mm^3^ unless they are vascularized, and tumors could be restricted to tiny sizes (110). New blood vessel formation from the existing vasculature establishment of a tumor blood supply plays a central role in distant metastasis in breast cancer (111, 112). A tumor cell secretes VEGF that interacts with VEGF receptors on the endothelial cell membrane and stimulates migration, proliferation, and neo-vessel formation from the adjacent established blood vessel (113). The VEGF family includes VEGF-A, VEGF-B, VEGF-C, VEGF-D, and placental growth factor (114). VEGF-A and its receptors VEGFR-1 and VEGFR-2 play major roles in pathological angiogenesis, including tumor angiogenesis, whereas VEGF-C/D and their receptor VEGFR-3 primarily function as critical regulators of lymphangiogenesis (113). A solid tumor microenvironment responds to low oxygen tension by enhancing the hypoxia-inducible factor (HIF) response (112). As a result, evidence showed that HIF-1α and HIF-2α activate several hypoxia-inducible gene pathways involved in angiogenesis and glycolysis (115, 116). On the other hand, activated NF-kB also promotes anti-apoptotic gene transcription (Bcl-2, Bcl-XL, and BCL-W) and proliferative gene expression (cyclin D) (14, 117, 118). In turn, Bcl-2 (sometimes called master regulators of anti-apoptosis) not only promotes cancer cell proliferation and invasion but also allows the chemo- and immunotherapeutic resistance of cancer cells (119). In this regard, apoptosis evasion *via* the over-expression of Bcl-2 or Bcl-XL has recently been proposed as a hallmark of cancer (120). A research conducted by Cochaud et al. explored that ER-negative BC is rich in increased infiltration of IL-17A-producing cells and PDL1 levels (28, 121). IL-17 promotes the expression of CCL17 and CCL22 and facilitates Treg cell migration to suppress antitumor immunity (122, 123). Thus, IL-17A also enhances proliferation and metastasis *via* inhibiting tumor apoptosis and suppressing antitumor immunity (53, 55, 121, 124, 125) [*i*.*e*., through decreasing CD4 T helper 1 (T_H_-1) cells and increasing Treg cell] (126).

## IL-17 signaling cascade as a therapeutic target of breast cancer metastasis

As elaborated well above, IL-17A is potentially significant in the growth, proliferation, and progression of human cancer, including breast cancer (28). Thus, in the animal model experiment, IL-17A is considerably a therapeutic target during the chemotherapeutic management of breast cancer since its inhibition decreases cancer progression, migration, and distant metastasis. As discussed earlier, IL-17A mediates cancer cell invasiveness and metastasis *via* MMP-2, MMP-9, and MMP-13. Furthermore, IL-17A stimulates MMP-9 mRNA expression, and MMP-9 inhibitors can inhibit the IL-17A-dependent invasion and metastasis of BCCs (17). The relation between IL-17A and its downstream MMP activity and breast cancer metastasis through MAPK and NF-Kb suggests the possibility of various strategies connected with blocking these checkpoints and kinase enzyme activity. Therefore, strategies target IL-17A by blocking downstream signaling molecules like MAPK or inhibiting specific gene products like MMPs. On the other hand, MMP can be inhibited simply by targeting the master transcription factor known as NF-kB.

### IL-17A as a potential therapeutic target

**Figure 4:**
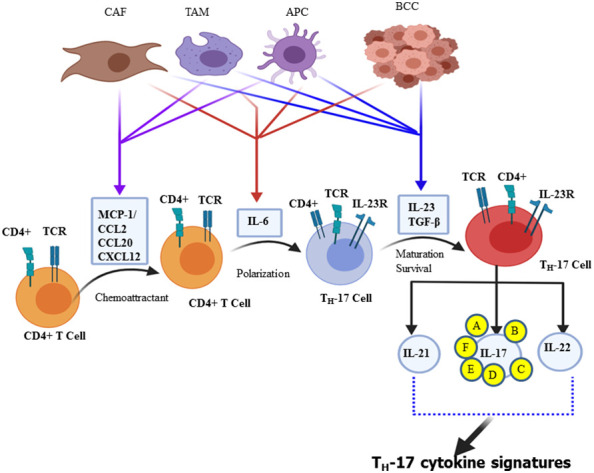
Schematic summary of the actions of various inhibitors of IL-17A and its multiple downstream signal transduction pathways. In addition to the synthetic and natural inhibitors of these signaling target molecules, extracellular signal-regulated kinase can be endogenously regulated by short negative feedback loop *via* dephosphorylation, dual-specificity phosphatase.

The role of IL-17 still has controversy and needs advanced research. IL-17-producing cells of both lymphocytic and myeloid origins or the microenvironment of the cancer cell as well as their suggested pro- and antitumorigenic functions in an organ-dependent context all contribute to make it purely a challenge to bring science to clinical practice (75). In support of this, a study showed the anti-tumorigenesis effects of IL-17E exposure to the breast cancer cell lines of MCF7, MDA-MB468, MDA-MB435-S,MDA-MB231, SKBR3,T47D, ZR75, Hs578t, HCC1937, and MDA-MB175-7 (**Table 1**) (34, 75). Th-17 cell infiltration with a common pro-inflammatory signature cytokine, IL-17A, is a crucial player in the proliferation, growth, migration, and dissemination of many cancer cells, including BC and many more types of cancer. **Table 1** shows a summary of the current correlation findings between different IL-17 subtypes producing T cells and their overall mechanism of pro-tumorigenesis in various breast cancer cell lines (75). As a result, research scholars understand the molecular mechanism of IL-17 in the development and progress of breast cancer and try to target the development of potential therapeutic options to tackle this life-threatening non-communicable disease. The Food and Drug Administration approved the fully-humanized anti-IL-17A monoclonal antibody secukinumab (AIN457) as an acceptable therapeutic choice for psoriasis, rheumatoid arthritis, ankylosing spondylitis, and other inflammatory diseases (**Figure 4**) (47, 127–129). Treating mice with ER- or triple-negative breast cancer by secukinumab boosts the antitumor immunity such as CD4+ and CD8+ T cells and decreases both the expression of PDL-1 and Treg cell infiltration (121). Interestingly, a combination treatment approach [anti-IL-17A (secukinumab) and anti-PDL1 (pembrolizumab)] improved antitumor immunity in support of its eradication (130).

### MAPK as a potential therapeutic target

Cochaud et al. reported that the IL-17A/IL-17AR interaction not only stimulates the proliferation and growth of the human BC cell line but also is responsible for chemoresistance (docetaxel). This pathologic mechanism of IL-17A was due to the upregulation of the ERK1/2 JNK and p38 MAPK pathway *via* MEK1/2 (69). Because p38 MAPK, ERK1/2, and MEK1/2 are the “Achilles heel” of tumor growth and cancer cell survival, targeting them helps negatively interrupt the typical proliferation environment of the cancer cell. The MEK1/2 inhibitor U0126 (IC50 = 0.5 uM) chemically inhibits MAPK signaling (131, 132). The ERK1/2 expression in *de novo* is negatively regulated by MAP kinase phosphatases (MKPs) or dual-specificity protein phosphatase through a negative feedback loop (133). In addition, ulixertinib, a reversible ATP-competitive small-molecule ERK1/2 kinase inhibitor, has shown promising results in phase 1 clinical trials (134). Furthermore, there is also a selective p38 MAPK α and β isoform inhibitor, ralimetinib (LY2228820 dimesylate), not only for BC patients but also for some other human cancer, including glioblastoma, multiple myeloma, ovarian, and lung cancer (**Figure 4**) (135, 136). The IC50 of LY2228820 for p38 alpha = 5.3 nM and for p38 beta = 3.2 nM. BIRB-796 (doramapimod) is also an inhibitor of p38 with IC50 for p38 alpha = 38 nM, for p38 beta = 65 nM, and for p38 delta = 520 nM (136).

### NF-kB as a potential therapeutic target

In mammals, there are five prominent member of the NF-κB family of transcription factors such as p50, p52, c-Rel, RelA (p65), and RelB (137). TRAF6 is the first signal transducer in NF-kB activation *via* IκB kinase (IKK) complex-mediated phosphorylation of the inhibitor of NF-κB proteins (IκBs) followed by ubiquitin–proteasomal degradation in response to IL-17. The IKK complex maintains NF-kB in the inactive state (NF-kB- IκB dimer) in the cytosol of unstimulated cells. Gene profiling analysis revealed that the constitutive activation of the NF-kB may be a key regulator (138) and a driving force for the pathogenesis of a variety of solid tumors, including BC and TNBCs (139). NF-κB is a set of transcription factors that play in various inflammation and immunity-associated diseases. It is also involved in different cancer progression and survival (140). The natural compound extracted from *Curcuma* and its isoxazole analog has many properties, such as anti-inflammatory and antitumor properties, especially TNBC cell lines and HL-60 in human leukemia by counteracting NF-kB activation (141).

Dehydroxymethylepoxyquinomicin (DHMEQ) is a synthetic and selective inhibitor of NF-kB at the site of its translocation (142, 143). Studies have shown that a substantial reduction of the activation of NF-kB is observed in TNBC patients presently treated with DHMEQ (142). Furthermore, dimethyl fumarate also effectively blocks NF-kB activity in multiple BCC lines (144). MG132 is another synthetic compound that targets and prevents the ubiquitin–proteasome degradation of NF-kB inhibitor, IkBα, or β (**Figure 4**) (145, 146).

### VEGF as a therapeutic target

Angiogenesis is essential for breast cancer progression and metastasis (147). The uncontrolled expression and activity of VEGF are very common in different cancer types, including BC, as reliable biomarkers for angiogenesis and vascularization (148). IL-17 induces the expression of specific chemokines like chemokine ligand 2 (CCL-2) and vascular endothelial growth factor (VEGF), which promotes IL-17-producing angiogenic macrophage, which can contribute to the microenvironment and angiogenesis (14). In support of this, a study conducted on the 4T1 BC cell line in the murine model explored that, with the administration of IL-17, VEGF mediated vasculogenesis and increased microvascular density (149, 150). The exuberant expression and circulatory detection of VEGF mRNA are predictors of poor prognosis factors (151). Thus, increased VEGF expression has been associated with poor response to tamoxifen or chemotherapy in patients with advanced breast cancer (111). Inhibiting or targeting VEGF is the most promising mode of chemotherapy for different types of solid tumors, including BC, and it also interrupts its metastatic ability. The monoclonal antibody drug bevacizumab targets and inhibits the activity of the soluble form of VEGF-A ligand due to the loss of its structural conformation. This results in inhibiting VEGF-mediated angiogenesis, metastasis, and tumor survival (148, 152). Therefore, bevacizumab led to a prominent prolongation in mean progression-free survival from 15.6 to 20.2 months (**Figure 4**) (153). Clinical and preclinical studies showed that exposure to trastuzumab significantly decreased VEGF in HER-2-overexpressing cells (154). Moreover, angiostatin is an endogenous inhibitor of angiogenesis or suppressor of neovascularization through negative inhibition of endothelial cell migration and proliferation. In turn, it augments tumor inhibition. Angiostatin is found naturally in humans and several other animals (152).

### MMP as a therapeutic target

MMPs are potential pharmacological therapeutic targets for treating invasive breast cancer (155). Endogenously, TIMP has natural and clinically significant therapeutic effects *via* inhibition of many MMPs. Rebimastat, an inhibitor of MMP-1, MMP-2, MMP-3, MMP-8, MMP-9, MMP-13, and MMP-14, significantly abolishes tumor growth and abrogates BC metastasis (156). Similarly, broad-spectrum batimastat has a broad-spectrum inhibition of virtually all MMP members (157, 158). In contrast, considering specificity and selectivity, anti-MMP-2 and anti-MMP-1—such as AG-3340, BAY 12-9566, and BMS-257291—and Ro 32-3555, respectively, are used as specific therapeutic options (155, 159). Furthermore, small molecules such as tanomastat, prinomastat, and rebimastat inhibit MMP-2, MMP-3, MMP-8, MMP-9, and MMP-13; MMP-2, MMP-3, MMP-9, MMP-13, and MMP-14; and MMP-1, MMP-2, MMP-3, MMP-8, MMP-9, MMP-13, and MMP-14, respectively (156, 158). In addition, a murine monoclonal antibody called REGA-3G12 inhibits MMP-9 without influencing the function of MMP-2 (**Figure 4**) (160).

## Conclusions and future direction

Breast cancer is still one of the leading threats to women’s life. In the stages of the disease, cancer can spread to distant organs, including the brain and bone, where chemotherapy is not easily accessible. Achieving effective cancer therapy is significantly hampered by inflammatory cancer microenvironments. Thus, targeting IL-17A signaling pathways provides a promising future approach to developing novel treatment options. Considering contradictory results observed in other research regarding the pro- and anti-cancer nature of the Th-17 cell, an individualized adjustment may be required with different cell lines and even in various stages of cancer to tackle or target the IL-17A downstream signaling axis. Targeting the IL-17/IL-17R axis in breast cancer as relayed in clinical and preclinical models surprisingly produces excellent outcomes due to the types of cell line stage of the disease and the exposure status of the cell with IL-17. The evidence suggested that targeting and reprogramming multiple downstream signaling pathways of IL-17A may be an essential complementary option to promote the efficacy of conventional chemotherapy to treat breast cancer metastasis. Therefore, further research is needed in the future to develop anti-cancer strategies that target IL-17 signatures and their signaling pathways.

## Author contributions

TS was involved in the conception, study design, execution, acquisition of data, analysis, and interpretation, and drafting and critical review of the paper. BT and BA were involved in literature search and drafting and critical review of the paper. All authors contributed to the article and approved the submitted version.
